# Molecular mechanisms involved in drug-induced liver injury caused by urate-lowering Chinese herbs: A network pharmacology study and biology experiments

**DOI:** 10.1371/journal.pone.0216948

**Published:** 2019-05-29

**Authors:** Fan Li, Yi-Zhu Dong, Dan Zhang, Xiao-Meng Zhang, Zhi-Jian Lin, Bing Zhang

**Affiliations:** Department of Clinical Chinese Pharmacy, School of Chinese Materia Medica, Beijing University of Chinese Medicine, Chao Yang District, Beijing, China; Rajendra Memorial Research Institute of Medical Sciences, INDIA

## Abstract

As an important part of the comprehensive treatment methods, the urate-lowering Chinese herbs could provide favorable clinical effects on hyperuricemia in its ability to invigorate spleen and remove dampness. Owing to the long-term duration, it brought up the potential adverse reactions (ADRs) and concerns about the drug-induced liver injury from these herbs. To address this problem, the bioinformatics approaches which combined the network pharmacology, computer simulation and molecular biology experiments were undertaken to elucidate the underlying drug-induced liver injury molecular mechanisms of urate-lowering Chinese herbs. Several electronic databases were searched to identify the potential liver injury compounds in published research. Then, the putative target profile of liver injury was predicted, and the interaction network was constructed based on the links between the compounds, corresponding targets and core pathways. Accordingly, the molecular docking simulation was performed to recognize the representative compounds with hepatotoxicity. Finally, the cell experiments were conducted to investigate the biochemical indicators and expression of the crucial protein that were closely associated with liver injury. In conclusion, the current research revealed that the compounds with potential liver injury including diosgenin, baicalin, saikosaponin D, tetrandrine, rutaecarpine and evodiamine from urate-lowering Chinese herbs, could lead to decline the survival rate of L-02 cell, increase the activities of aspartate aminotransferase (AST), alanine aminotransferase (ALT), lactate dehydrogenase (LDH) and alkaline phosphatase (ALP) in cell-culture medium, enhance the expression of p-p38/p38, while the p38 inhibitor could achieve the trend of regulating and controlling liver injury. These research findings bring further support to the growing evidence that the mechanism of the liver injury induced by the compounds from urate-lowering Chinese herbs may be associated with the activation of p38α.

## Introduction

Hyperuricemia is defined as a serum urate concentration exceeding the limit of solubility (approximately 6.8 mg/dl), is commonly considered as a metabolic abnormality that caused by the obstacles in purine metabolism or a decrease in the excretion of uric acid [1–3]. In view of the rapid economic development along with the diet and lifestyle changes, the prevalence of hyperuricemia has increased over 21% and 13% in the United States and Chinese general populations, respectively. Therefore, there has been an increasing trend in the prevalence of hyperuricemia, it becomes a serious public health problem worldwide currently [4–8]. Furthermore, hyperuricemia has been viewed as a clinically important risk factor for various diseases by numerous epidemiological studies, the higher level of serum uric acid are involved in metabolic syndrome (including hypertension, obesity, type 2 diabetes, dyslipidaemias, etc.), renal impairment (including chronic kidney disease, etc.), cardiac diseases (including coronary heart disease, heart failure and atrial fibrillation), stroke and peripheral arterial disease [9–13]. Recently, non-steroid anti-inflammatory drugs, benzbromarone, and allopurinol have a rapid onset for urate-lowering, making it a popular choice for the treatment of hyperuricemia [14–17]. Nevertheless, with the long-duration of hyperuricemia, these agents are associated with ADRs, for example, treatment with benzarone or benzbromarone may have the detrimental impact on hepatic injury [18]. And it is reported that allopurinol could cause hypersensitivity syndrome or Steven-Johnson syndrome in some cases [19].

To this relevant issue, traditional Chinese Medicine (TCM) has such advantages as multiple pathways, multi-targets to lower serum uric acid levels, and it has been extensively used in clinical practice for the thousands of years in Asian countries [20–21]. Theoretically speaking, Chinese herbs can exert the therapeutic effects of invigorating spleen and kidney, removing dampness and clearing away turbidness for regulating the uric acid metabolism; and it is regarded also as a considerable treatment by improving the metabolism of blood lipid abnormality and hepatorenal function [22–23]. Functionally, the candidate targets of TCM are significantly associated with several biological pathways by molecular biology techniques, such as regulating the mRNA and protein expressions of uric acid transporter and inhibiting the activity of xanthine oxidase [24–25]. However, given controlling the level of serum uric acid is a long-term process; therefore, it would also bring up the ADRs owing to the long duration of Chinese herbs. Recent pharmacological studies have declared that some herbal ingredients or compounds for treating hyperuricemia might improve the risk of ADRs, it is demonstrated that kaempferol and thymol in *Xiaochaihutang* and rhein in *Heshouwu* may be related to the potential liver injury targets [26–27]. With regard to the underlying drug-induced liver injury, the molecular mechanisms of urate-lowering Chinese herbs have not been fully elucidated due to the lack of appropriate research approaches. We performed the relative mechanism analysis via the approaches of network pharmacology, molecular docking simulation and cell experiments at the molecular level, which are the powerful tools based on contemporary in silico and in vitro methods to elucidate holistic and complex mechanisms of TCM with the rapid progress of bioinformatics, systems biology, and polypharmacology.

## Materials and methods

**Fig 1:**
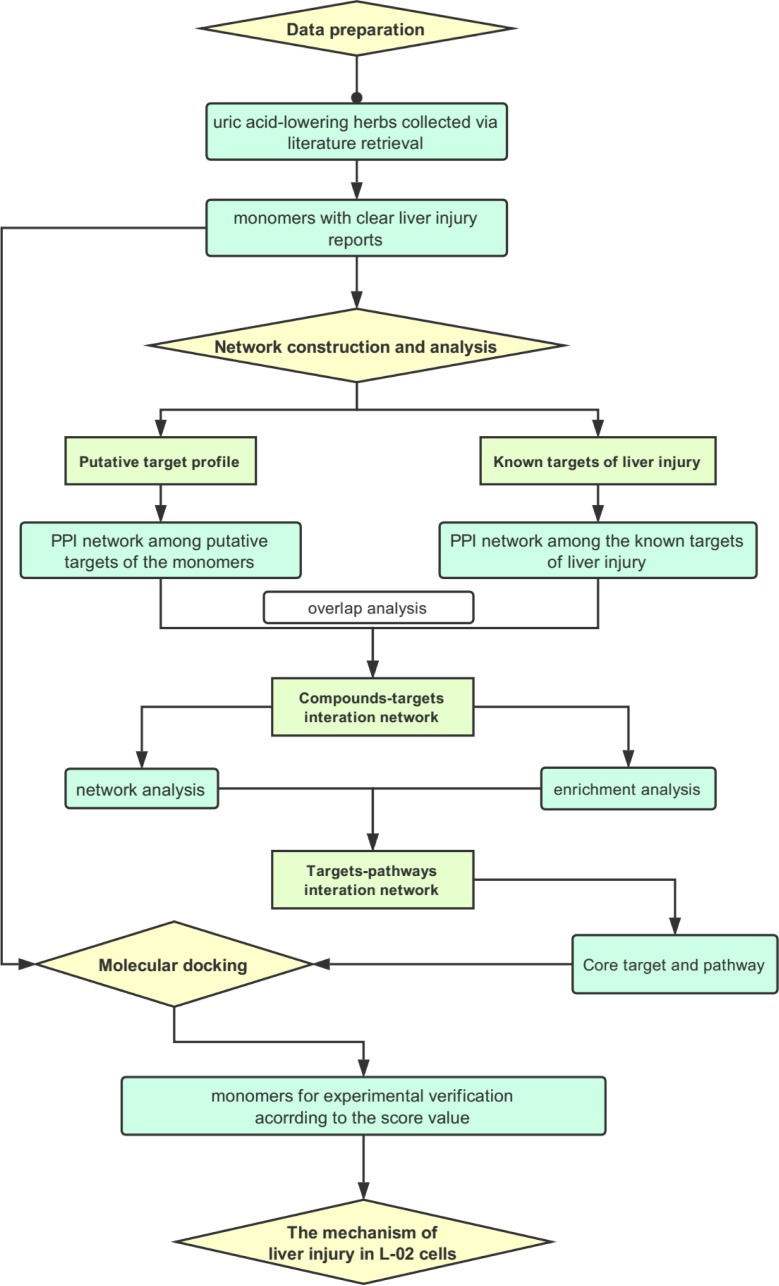
The flowchart of technical strategy in current study.

The current study was performed by four-step analysis, namely data preparation, network construction and analysis, molecular docking simulation and cell experiment in vitro. And the flowchart of the technical strategy was presented in Fig 1.

### Collection of potential drug-induced liver injury components in urate-lowering Chinese herbs

First, all kinds of studies concerning Chinese herbs that can induce drug-induced liver injury were selected by searching the following electronic databases up to May 2017: PubMed, Embase, Springer, the China National Knowledge Infrastructure Database (CNKI), the Wanfang Database, the Chinese Scientific Journals Full-text Database (VIP), and the Chinese Biomedical Literature Database (SinoMed). Moreover, according to the information of Chinese herbs that were recorded in the textbook of *Chinese Materia Medica* and Chinese pharmacopeia [28–29], the searching terms of Chinese herbs included the Decoction pieces name, Latin name, and *pinyin*. And the searching terms of drug-induced liver injury covered “adverse drug event*”, “side effect*”, “adverse drug reaction*”, “drug toxicit*”, “toxicity”, “toxic potential”, “liver injury”, “hepatotoxicity, “liver damage”. Second, the literature with repetitive content, efficacy studies, the ADR of TCM formulas or Chinese patent medicine, and ADRs without liver injury was excluded via NoteExpress software. Through comprehensive collecting and combining the published literature of liver injury induced by urate-lowering Chinese herbs, the compounds or ingredients with potential liver injury from urate-lowering Chinese herbs were identified to build the database. Third, the Chem 3D package of molecular modeling software Chemoffice 2004 was utilized to depict the chemical and crystal structures, calculate various thermodynamic, electronic or steric parameters, and optimize molecular force field, ultimately, these data of components with potential liver injury was saved as mol 2 format [30–31].

### Prediction of putative liver injury targets

On the one hand, the related targets corresponding to the selected components were acquiring from traditional Chinese medicine systems pharmacology database and analysis platform (TCMSP) (http://lsp.nwu.edu.cn/tcmsp.php) and PharmMapper Database (http://lilab.ecust.edu.cn/pharmmapper/) [32–33]. It was noteworthy that TCMSP was developed as a digital repository of TCM, with more than 30,000 ingredients of Chinese herbs retrieved through literature mining and database integration [34]. In addition, Pharm Mapper Database was an online tool for drug-target identification via pharmacophore mapping approach [35]. During this procedure, “Human protein targets only (v2010, 2241)” was selected, besides, other parameters were set to default values, and the targets with z’-score>0 of components with potential liver injury were chosen. The related targets from TCMSP and Pharm Mapper Database was in standard processing under the universal protein knowledgebase—UniProt (https://www.uniprot.org/) [36], the database of targets corresponding to the 32 candidate components with liver injury was built after identifying the redundant proteomes. On the other hand, the candidate target proteins relating to liver injury (keywords: “drug induce”, “drug-induced”, “drug-induced liver injury”, “drug-induced chronic hepatitis”, “liver injury, drug-induced”, “hepatitis, toxic”, “toxic liver disease”) were acquired in the Online Mendelian Inheritance in Man (OMIM) database (http://www.omim.org/, updated on April 21, 2016) [37], The Therapeutic Target (TTD) Database (http://bidd.nus.edu.sg/group/cjttd/,updated on Sep 10th, 2015) [38], the Pharmacogenomics Knowledgebase (PharmGKB) Database (http://www.pharmgkb.org) [39] and the Genetic Association Database (GAD) (https://geneticassociationdb.nih.gov/,updated on August 18, 2014) [40]. After removing redundant entries, the remaining proteins relating to liver injury were considered as candidate targets. Afterwards, the overlapping targets for the database of targets corresponding to the 32 candidate components from TCMSP and Pharm Mapper Database, and database of liver injury proteins from OMIM, TTD, PharmGKB, GAD were gathered as putative liver injury targets of 32 candidate components in current study for subsequent analyses.

### Protein-protein interaction (PPI) data

Further, to identity hub genes which were involved in pathogenesis of liver injury at the protein interaction level, protein-protein interaction (PPI) data were derived from the Cytoscape plugin Bisogenet [41], and analyzed based on the six existing PPI databases including IntAct molecular interaction database (https://www.ebi.ac.uk/intact/) Human Protein Reference Database (http://www.hprd.org/) [42–43], Molecular interaction Database (https://mint.bio.uniroma2.it/) [44], Database of Interacting Proteins (https://dip.doe-mbi.ucla.edu/dip/Main.cgi) [45], Biological General Repository for Interaction Datasets (https://thebiogrid.org/) [46] and Biomolecular Interaction Network Database (http://bind.ca) [47]. Remarkably, Bisogenet was accepted as a multi-tier application for visualization and analysis of biomolecular relationships in the field of bioinformatics due to it can integrate data from different sources in a fast and user-friendly manner, in addition, provide the suitable framework and flexible approaches to reconstruct, represent and analyze topological networks for PPI data [48].

### Network construction and analysis of components with potential liver injury

First, the components-targets (C-T) network was constructed by the interaction of the candidate compounds with the corresponding proteins that obtained from the above mentioned Cytoscape plugin Bisogenet. The topological features such as degree, betweenness, and closeness, were used to select the putative targets by means of Cytosacape plugin CytoNCA [49]. The twofold median value of node degree, the onefold median value of betweenness and closeness were applied as a cutoff point in present network. The degree of a node was defined as the number of edges connecting to a node, and the betweenness centrality was corresponded to the frequency with which shortest paths between any pair of nodes, and the closeness centrality was measured the importance of a node in a subnetwork [50]. The definitions and computational formulas of these parameters were previously defined and represented the topological importance of a node in the network. After predicting with the three parameters, the component-target interaction network was thereafter visualized with Cytoscape program (Version 3.2.1) [51]. Second, the targets-pathways (T-P) network was built through the connection of the targets and their own pathways, respectively. In addition, the pathway enrichment analysis of the candidate targets was accessible from the DAVID Bioinformatics Database (Resources 6.8, http://david.abcc.ncifcrf.gov), a web-based online bioinformatics resource that aimed to provide tools for the functional interpretation for large lists of genes or proteins [52]. The core pathway was screened in Omicshare 3.0 (http://www.omicshare.com/). Also, the pathway was annotated by employing the Reactome Pathway Database (http://www.reactome.org), which was an open source, expert-authored, peer-reviewed, manually curated database of reactions, pathways and biological processes [53]. Based on the C-T network and T-P network, the core target and pathway of liver injury for candidate components would be considered as the receptor protein in following molecular docking simulation.

### Molecular docking simulation

The molecular docking simulation was performed to verify the binding affinity of candidate compounds and liver injury core target [54]. First, Protein Data Bank (PDB) database (http://www.rcsb.org/pdb/home/home.do, updated on March 11, 2014) was used for retrieving the protein conformation of core target with liver injury, of *Homo sapiens origin* [55], meeting following criterion was recognized as appropriate protein conformation: 1)The three-dimensional protein structures were determined via X-ray crystallography; 2) The crystal resolution of protein was smaller than 3Å; 3) The protein analysis of genotype was definite and reliable; 4) The protein conformation that had been proven and supported by published information was given the priority. Second, directly downloaded the crystal structures of the candidate targets as the file format called pdbqt from the PDB database, and the process of adding hydrogen atoms and charges, merging non-polar hydrogens bond identification and selector statics calculations, predicting binding site and ligand deletion, superimposing homologous or mutant structures, and testing conformational changes were presented through the UCSF Chimera (www.cgl.ucsf.edu/chimera) [56–57] and Autodock Tool 4.0 [58–59]. Third, the rectangular boxes for the definition of binding site were predicted, the binding poses and grid box of compounds were assessed; molecular docking among candidate compounds and core target for liver injury was conducted by Autodock Vina [60–61]. Furthermore, the docking poses were ranked according to their docking scores, and the compounds with higher binding affinity were selected to illustrate their corresponding binding poses and sites via Pymol software [62]. In addition, different hydrogen-bond and hydrophobic interactions of ligand-binding sites were displayed through Ligplus software [63]. The toxic mechanism of representative compounds with higher binding affinity was explored via the following cell experiment.

### Cell culture

The normal hepatic cell (L02) cell lines were obtained from the National Infrastructure of Cell Line Resources (Chinese Academy of Medical Sciences, Beijing, China), and maintained in Dulbecco’s modified Eagle’s medium (DMEM, 10-013-CVR) supplemented with 10% fetal bovine serum (FBS, Hyclone), 1% penicillin and streptomycin (PS, 100 IU/ml penicillin, 100 μg/ml streptomycin, Mediatech, 30-002-CI). All cells were incubated in a humidified atmosphere at 37°C with 5% CO_2_.

### MTT assays

L-02 cells were trypsinized by 0.25% trypsin after reaching the confluence of 70–80% and plated in 96-well plates at the density of 1.6 × 10^3^ cells/plate. And L-02 cells were cultured for 24 hours in vitro before exposure to varying concentrations of different representative compounds with liver injury for further incubating in another 24 hours. Then, 20 μl of 0.25 mg/ml3-(4,5-dimethylthiazol-2-yl)-2,5-diphenyltetrazolium bromide (MTT)-tetrazolium salts (Sigma-Aldrich Corp., St. Louis, MO, USA) in PBS was added to each hole in 96-well plates. After 4 hours of incubation, the formazan crystals were dissolved by 150μl Dimethyl Sulphoxide (DMSO), and shocked to dissolve thoroughly, then the cell viability was measured by MTT method at 570 nm [64–65]. All experiments were repeated in triplicate independently.

### Biochemical indicators of hepatotoxicity in cell

To determine how potential liver injury-compounds affected biochemical indicators of L-02 cells, we investigated the activities of AST, ALT, LDH and ALP in cell migration capacity under the different representative compounds conditions (representative compounds group), the inhibitor conditions (inhibitor group), and the negative control. For these experiments, three replicates were made for each concentration of the different representative compounds with liver injury, and each group was established three holes and cultured in 6 times repeatedly. L02 cells were settled into 24-well plate at concentrations of 1.6 × 10^3^ per well for 24h and then treated with the concentrations that had been determined by MTT method of the representative candidate liver injury compounds for another 24 h. Subsequently, the supernatant extraction of L02 cells was collected and centrifuged to detect the activities of AST, ALT, LDH, and ALP by chromatometry method.

### Western blot

After the examination of biochemical indicators for hepatotoxicity in the cell, the expression of crucial liver injury protein in L02 cell lines among different groups was compared by Western blot analysis. Briefly, the proteins from different groups were harvested and lysed using lysis buffer (Sigma-Aldrich) containing a proteinase inhibitor cocktail (Sigma-Aldrich), the equal amounts of protein were separated by electrophoresis on a pre-cast 10% SDS-polyacrylamide gel (Bio-Rad, Hercules, CA) and transferred to polyvinylidene difluoride membranes (Millipore, Bedford, MA). Next, the membrane was blocked in TBST-T containing 5% skim milk for 2 h at room temperature and probed with anti-beta-catenin, and incubated overnight with the indicated primary antibody, followed by incubation with the secondary antibodies at room temperature for another 2 h. Finally, all the immune blots were visualized by enhanced chemiluminescence (Pierce Biotechnology, Inc., Rockford, IL, USA) and western blots were performed at least three times.

### Statistical analysis

The values were presented as the mean ± standard deviation (SD) from 3 independent experiments for 4 cell lines. The statistical analysis was performed with unpaired t-test and one-way ANOVA by GraphPad Prism 5 software (GraphPad Software, Inc, La Jolla, USA), and p < 0.05 was considered to be statistically significant.

## Results

### The potential liver injury components of urate-lowering Chinese herbs

Initially, 61,236 published articles concerning drug-induced liver injury were retrieved according to above-described search strategy, a total of 171 urate-lowering Chinese herbs were collected and enrolled through the comprehensive retrieval in the present study. Additionally, 7 individual herbs exerted the obvious effects of urate-lowering, the compatibility of 134 herbs in formulas presented a synergistic combination for treating hyperuricemia, and 30 herbs against hyperuricemia can be used both alone and compatible. Through further inspection and excluded irrelevant literature, ultimately 481 articles that reported 32 candidate components from urate-lowering Chinese herbs were enrolled in present research, these components were associated with liver damage and hepatic metabolism hindrance. Remarkably, the majority of them were the simultaneously effective and toxic constituent in herbs, and the details about the component with liver injury from urate-lowering Chinese herbs were provided in Table 1.

**Table 1:** The details about the components with liver injury from urate-lowering Chinese herbs.

Number	Components	Structural formula	Molecular mass
1	Arecoline	C_8_H_13_NO_2_	155.20
2	Saikosaponin D	C_42_H_68_O_13_	780.47
3	Dioscin	C_45_H_72_O_16_	869.06
4	Bergapten	C_12_H_8_O_4_	216.20
5	Xanthotoxin	C_12_H_8_O_4_	216.19
6	Osthole	C_15_H_16_O_3_	244.29
7	isopsoralen	C_11_H_6_O_3_	186.17
8	Tetrandrine	C_38_H_42_N_2_O_6_	622.76
9	Puerarin	C_21_H_20_O_10_	432.38
10	Berberine	C_20_H_18_ClNO_4_	371.82
11	Baicalin	C_21_H_18_O_11_	446.36
12	Baicalein	C_15_H_10_O_5_	270.24
13	Curcumin	C_21_H_20_O_6_	368.38
14	Sophocarpine	C_15_H_22_N_2_O	246.35
15	Sophoraflavanone G	C_25_H_28_O_6_	424.49
16	Matrine	C_15_H_24_N_2_O	248.37
17	Kurarinone	C_26_H_30_O_6_	438.52
18	Oxysophocarpine	C_15_H_22_N_2_O_2_	262.35
19	Oxymatrine	C_15_H_24_N_2_O_2_	264.37
20	Gentiopicroside	C_16_H_20_O_9_	356.33
21	Reynosion	C_15_H_20_O_3_	248.32
22	Santamarine	C_15_H_20_O_3_	248.32
23	Aucklandiae	C_15_H_24_O	220.00
24	Elemol	C_15_H_26_O	222.37
25	Dehydrocostuslactone	C_15_H_18_O_2_	230.31
26	Artesunate	C_19_H_28_O_8_	384.42
27	Colchicine	C_22_H_25_NO_6_	399.44
28	Rutaecarpine	C_18_H_13_N_3_O	287.32
29	Evodiamine	C_19_H_17_N_3_O	303.37
30	1-Tetrahydropalmatine	C_21_H_25_NO_4_	355.43
31	Genipin	C_11_H_14_O_5_	226.23
32	Geniposide	C_17_H_24_O_10_	388.37

### The construction and analysis of network pharmacology

**Fig 2:**
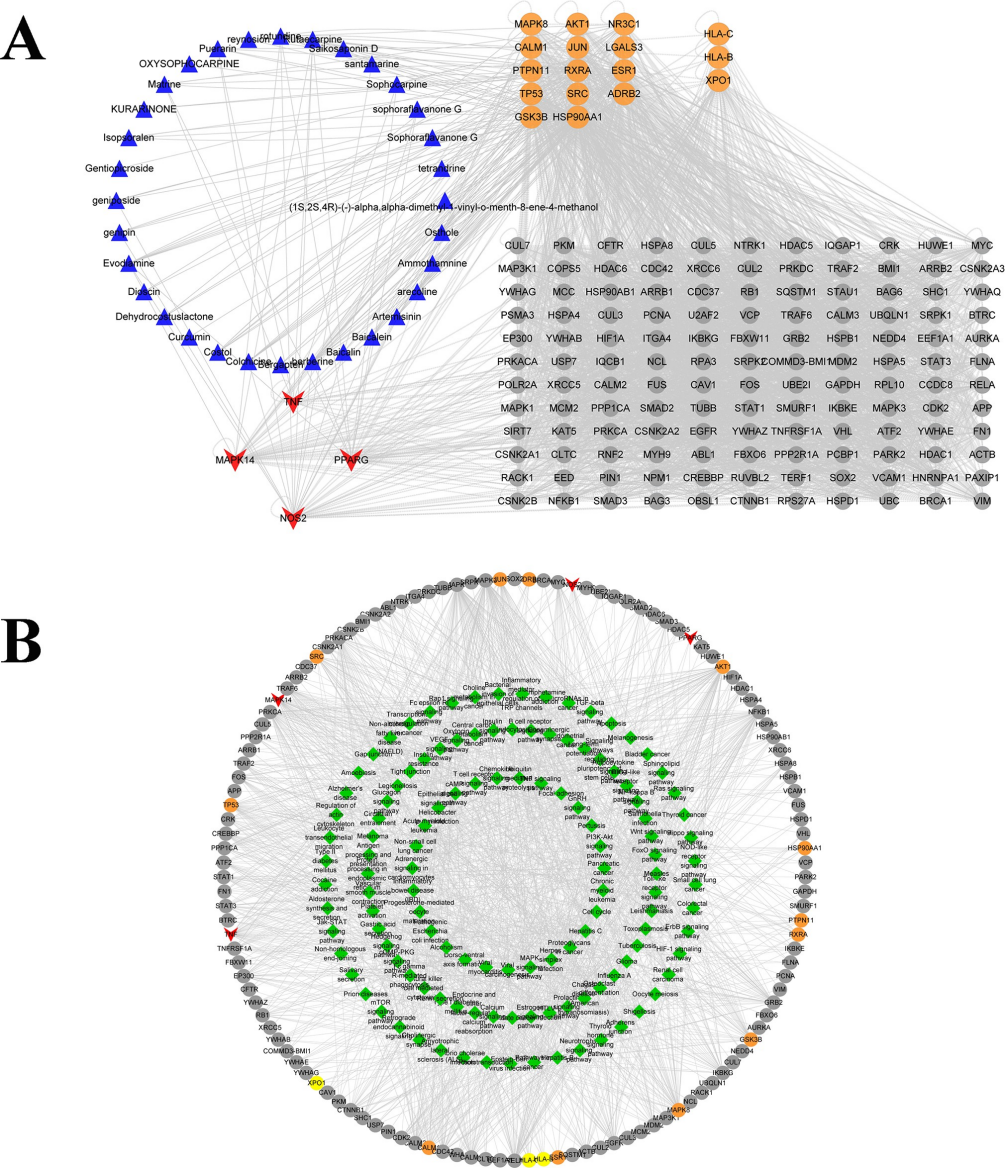
Component-target interaction network and target-pathway network. Note: (A.) The potential liver injury components urate-lowering Chinese medicine are indicated by blue triangles, the core targets are indicated by red arrows; the direct targets of components and “liver injury” are represented by yellow circles, and the interaction targets of components and “liver injury” are gray. (B.) The pathways are represented by green diamond. The targets are indicated by red arrow; the direct target of the compound and disease are represented by yellow circle, and the interacting proteins are represented by gray circle. To get an initial sense of the biological processes and pathways enriched by putative targets that were relative with liver injury, the functional enrichment analysis was conducted based on DAVID. And the results were classified into several signaling pathways, including pathways in cancer, Epstein-Barr virus infection, Viral carcinogenesis, p38α mitogen-activated protein kinase (MAPK) signaling pathway, the immune system, signal transduction, gene expression, cell cycle, DNA replication, and other processes in Fig 2.

Totally, we collected 272 targets corresponding to the components with potential liver injury through the aforementioned databases, PPI networks were constructed for the putative targets that were related to 32 components with liver injury. Then, the putative C-T interaction network of liver injury consisted of 1790 nodes. Following the construction of the C-T interaction network and the calculation of three topological features (degree, betweenness, and closeness) for each core target, the screening of candidate targets was performed under the conditions of degree >29, closeness >0.43523, betweenness >0.00015, using the twofold median value of node degree, the onefold median value of betweenness and closeness in this network as a cutoff point, 153 nodes were identified as core targets including mitogen-activated protein kinase 14 (MAPK14), nitric oxide synthase and inducible (NOS2), peroxisome proliferator-activated receptor gamma (PPARG), and tumor necrosis factor (TNF). The detailed information on the core targets in the C-T interaction network was presented in Table 2, the component-target interaction network and target-pathway network was illustrated in Fig 2. The information of top 10 pathways was shown in Table 3.

**Table 2:** The detail about core target in the component-target interaction network.

Target	Degree	Betweeness	Closeness
MAPK14	237	0.028579	0.506369
NOS2	157	0.006274	0.470542
PPARG	155	0.01105	0.499024
TNF	116	0.019764	0.44151

**Table 3:** The detail of the top 10 pathways in the target-pathway interactive network.

Pathway	Target hit quantity	P-value
Pathways in cancer	42	4.33E-20
Epstein-Barr virus infection	39	4.55E-29
Viral carcinogenesis	31	8.26E-19
MAPK signaling pathway	30	3.88E-15
Hepatitis B	28	9.02E-20
PI3K-Akt signaling pathway	28	2.96E-10
Herpes simplex infection	26	5.44E-15
HTLV-I infection	26	1.26E-11
Proteoglycans in cancer	25	3.83E-13
Neurotrophin signaling pathway	23	4.44E-16

**Fig 3:**
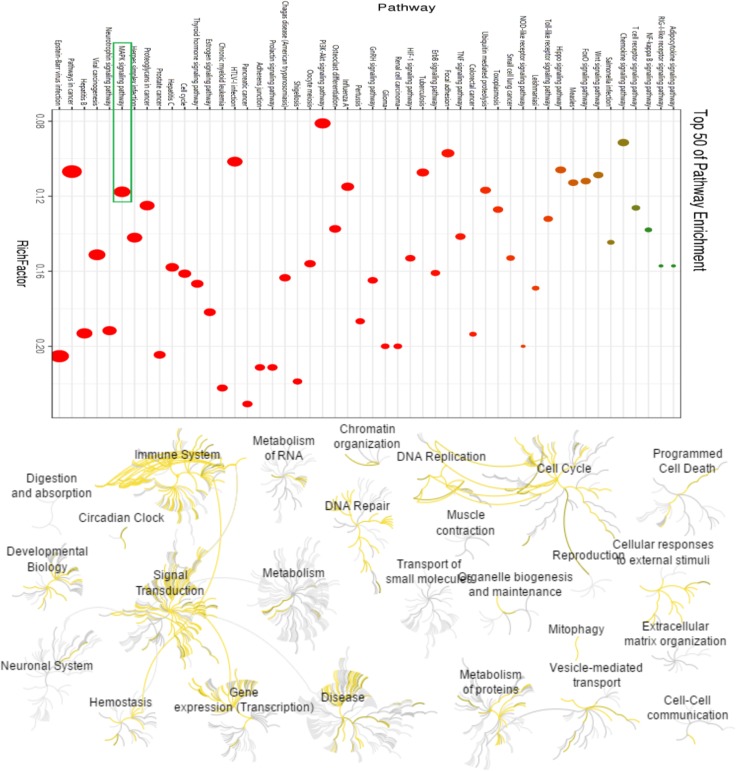
Distribution and the bubble map of core pathways. Note: (A.) The vertical axis is the path name and the horizontal axis is the Rich Factor value. The larger the P-value, the higher the channel enrichment; the size of the point indicates the number of enriched targets; the color of the dots is red to green. The value is from small to large. (B.) The yellow to brown pathway lines represent the pathways for important target enrichment, from yellow to brown indicating that the pathway P-values are small to large.

As shown in Fig 3, the biological processes by the MAPK signal pathway were often associated with the higher target hit quantity. Especially, the MAPK signal pathway had been indicated to contribute to liver metabolic changes, liver steatosis, and hepatic cell apoptosis during the progression of gluconeogenesis and lipid metabolism. As the major p38 MAPK isoform, p38α ubiquitously expressed at high levels in most cell types and played a pivotal role in elevating liver enzymes, survival genes, DNA damage, oxidative stress, inflammation response due to negatively regulate the G1/S and G2/M cell cycle transitions [66–68]. Accordingly, p38α was chosen as the core target for further elucidating the related mechanisms of liver injury in following molecular docking simulation.

### Molecular docking simulation

**Fig 4:**
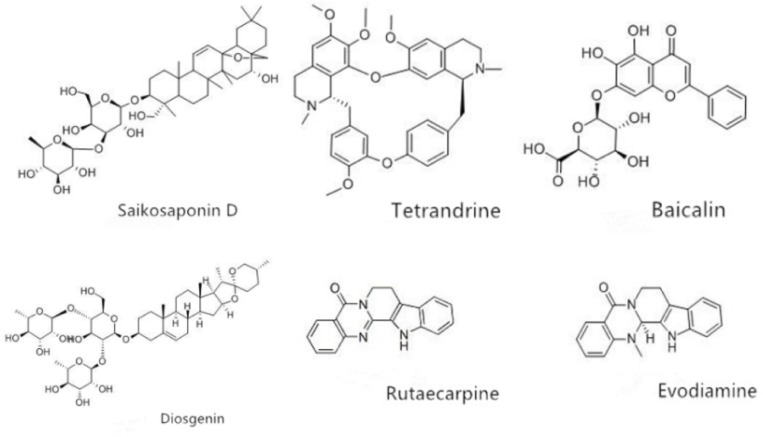
The structure of 6 compounds with strong binding capacity for p38.

**Fig 5:**
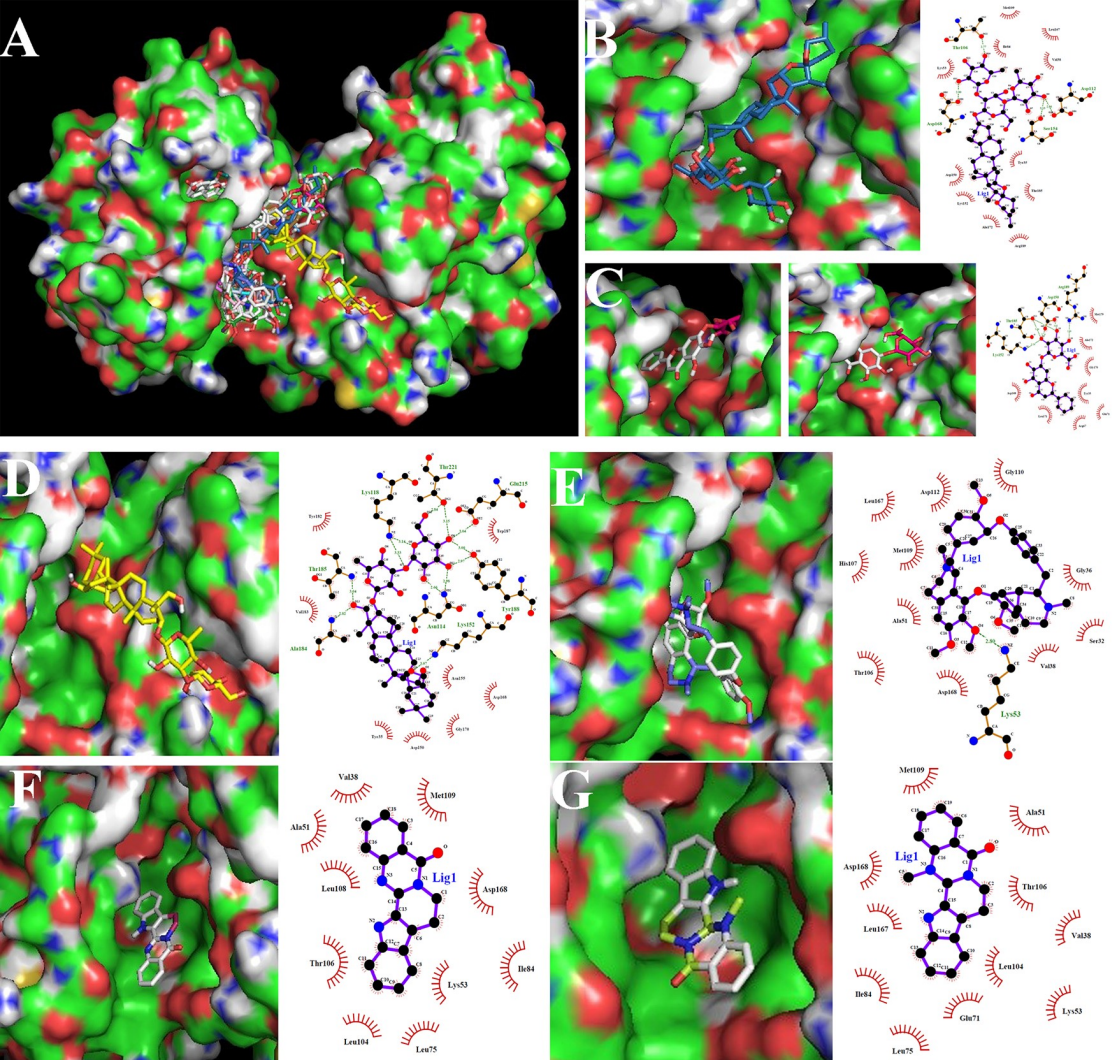
Docking modes between potential liver damage components and p38. Note: (A.) 32 potential liver injury components have entered the p38α protein active pocket. (B.) The small molecule of diosgenin penetrated into the active pocket, and mainly forms four hydrogen bonds with the Thr106, Asp168, Asp112 and Ser154 residues on the protein, atotal of 15 residues were bound by hydrophobic interaction on the p38α. (C.) The baicalin small molecule completely penetrated into the active pocket, forming 11 hydrogen bonds with the Arg189, Thr185, Asp150, Lys152, Tyr35 residues on the protein, and the sugar moiety contributed 9 hydrogens. The aglycone part contributed 2 hydrogen bonds, and more hydrophobic interaction with the protein. The hydrophobic interaction residues of the baicalin molecule were 13. (D.) The saikosaponin D molecule completely entered the active pocket, mainly with Glu215 and Thr221 on the protein, Tyr188, Asn114, Lys118, Ala184, Thr185, Lys152 residues form 13 hydrogen bonds, a total of 16 hydrophobic residues. (E.) Tetrandrine small molecules deeped into the active pocket, forming a hydrogen bond with Lys53, and 13 residues formed a hydrophobic effect. (F.) Rutaecarpine completely entered the active pocket and formed a hydrophobic interaction with 10 residues. (G.)Evodiamine completely entered the active pocket, and 11 residues formed a hydrophobic effect.

In present work, molecular docking was carried out between all of the 32 candidate compounds with liver injury and p38α. To validate the docking method and docking accuracy, the different compounds was docked into the binding site of p38α, respectively. Both the ligand and receptor were isolated from the complex crystal structure in the PDB database. The results indicated that there were 6 representative compounds with an acceptable reliability of the docking method (binding free energy≤-8 kcal/mol) for the p38α, and these compounds included diosgenin, baicalin, saikosaponin D, tetrandrine, rutaecarpine, and evodiamine, besides, the structures of these 6 representative compounds with strong binding capacity for p38αwere depicted in Fig 4, the information of binding free energy for 32 potential liver injury components was shown in Table 4. Moreover, the main binding mode between the pairs of p38α and compounds was hydrogen bonding based on the analysis of Pymol and Ligplot, the 6 representative compounds with strong binding capacity were taken as the examples, the docking results between these compounds and p38α were shown in Fig 5. The molecular docking results laid the material foundation for the observation of biological effects subsequently.

**Table 4:** Binding free energy for 32 components with potential liver injury.

Number	Components	Binding free energy (kcal/mol)	Number	Components	Binding free energy (kcal/mol)
1	Diosgenin	-8.9	17	Santamarine	-6.8
2	Baicalin	-8.3	18	1-Tetrahydropalmatine	-6.8
3	Saikosaponin D	-8.2	19	Gentiopicroside	-6.7
4	Tetrandrine	-8.2	20	Dehydrocostuslactone	-6.7
5	Rutaecarpine	-8.2	21	Reynosion	-6.7
6	Evodiamine	-8	22	Sophocarpine	-6.6
7	Kurarinone	-7.9	23	Xanthotoxin	-6.6
8	Sophoraflavanone G	-7.6	24	Aucklandiae	-6.5
9	Artemisinin	-7.3	25	Matrine	-6.5
10	Puerarin	-7.2	26	Ammothamnine	-6.5
11	Baicalein	-7.2	27	isopsoralen	-6.4
12	Berberine	-7.1	28	Osthole	-6.1
13	Curcumin	-7	29	Bergapten	-5.9
14	Genipin	-6.9	30	Geniposide	-5.4
15	Colchicine	-6.9	31	Elemol	-5.3
16	Oxysophocarpine	-6.9	32	Arecoline hydrobromide	-4.2

### Evaluation of hepatotoxicity for representative components with liver injury

The hepatotoxicity for representative components from urate-lowering Chinese herbs was evaluated by investigating on L-02 by means of incubating with various concentrations of different components. The results of MTT assay demonstrated that diosgenin, rutaecarpine and evodiamine could achieve the good potency inhibitor against the cell viability of L-02 at a dose of 5μmol/L. Tetrandrine and saikosaponin D were associated with the lower cell survival rate at the dose of 60–90 μmol/L. These results of biochemical analyses were consistent with the findings of previous network pharmacology. Interestingly, baicalin had the trend of proliferative effects on L-02 at the dose of 2000μmol/L, indicating this compound may not cause liver cell injury compared to others. In accordance with the results of cells, the order of hepatotoxicity by potential liver injury components may be diosgenin, rutaecarpine, evodiamine, tetrandrine, saikosaponin D and baicalin. The specific composition information was shown in S1 Table. In each of the drug-administered groups, three concentrations before and after the cell viability significantly decreased were subjected to subsequent experiments.

The results of the colorimetric assay for investigating ALT, AST, LDH, and ALP activities among different components groups, revealed that the levels of ALT, AST, LDH and ALP increased obviously in potential liver injury components groups, suggesting that these components could exert hepatotoxicity in L-02 cells. In regard to baicalin, the baicalin group 1000μmol/L were associated with higher levels of ALT, AST, LDH and ALP than the blank group and the difference between both groups was not statistically significant, while the dose of 2000μmol/L could significantly increase the levels of ALT, AST, LDH and ALP than the blank group. Thereby baicalin could inhibit the proliferation of L-02 cells instead of inducing the cell damage. The specific composition information was shown in Table 5.

**Table 5:** ALT, AST, LDH, ALP enzyme activity of L-02 cells interfered with the representative components with liver injury from urate-lowering Chinese herbs.

Group		ALT(U/L)	AST(U/L)	LDH(U/L)	ALP (King's unit /100ml)
Dioscin	Blank	5.81±0.23	360.61±16.38	1.79±0.25	16.72±0.90
	Control	7.28±1.21	533.67±35.52**	1.84±0.09	17.30±1.02
	1μmol/L	7.53±0.61**	523.02±30.77**	1.58±0.39	16.03±1.60
	5μmol/L	6.88±0.50**	524.30±19.64**	3.67±0.30**^ΔΔ^	15.58±1.17
	10μmol/L	7.53±0.11**	561.38±74.95**	4.58±0.64**^ΔΔ^	15.91±0.48
Baicalin	Blank	1.52±0.80	502.37±50.60	1.03±0.24	1.55±0.19
	1000μmol/L	4.45±1.09*	690.76±33.24**	0.81±0.10	2.03±0.44
	2000μmol/L	4.48±0.13**	824.64±39.22**	0.84±0.18**	1.94±0.21*
Saikosaponin D	Blank	8.00±2.94	382.43±40.86	1.32±0.05	15.78±1.68
	Control	7.74±1.64	611.11±60.86**	1.13±0.12*	15.83±0.82
	50μmol/L	7.75±1.25	560.72±37.00**	1.13±0.25	15.58±1.07
	70μmol/L	14.41±0.60**^ΔΔ^	1472.87±52.32**^ΔΔ^	0.95±0.17**	23.12±2.09**^ΔΔ^
	90μmol/L	16.00±0.74**^ΔΔ^	1497.42±60.20**^ΔΔ^	1.00±0.08**	22.19±1.23**^ΔΔ^
Tetrandrine	Blank	3.43±0.21	589.59±69.45	0.92±0.10	14.36±0.82
	Control	4.52±0.80*	577.48±56.62	0.98±0.20	15.00±0.60
	40μmol/L	4.36±0.35*	713.48±49.44*^Δ^	0.79±0.08	16.55±0.71*^Δ^
	60μmol/L	5.01±1.20*	705.81±98.97^Δ^	0.82±0.16	16.39±0.81*
	80μmol/L	4.84±0.86*	736.08±77.41*^Δ^	0.93±0.07	17.45±0.35**^ΔΔ^
Rutaecarpine	Blank	2.66±0.99	588.31±96.50	1.08±0.14	2.25±0.48
	Control	9.42±1.62**	1072.79±58.59**	1.76±0.50*	5.86±0.09**
	5μmol/L	9.90±0.99**	1075.18±91.40**	1.62±0.04**	5.77±0.54**
	10μmol/L	8.81±1.85**	1127.68±56.72**	1.54±0.36*	5.67±0.12**
	15μmol/L	9.33±1.85**	1000.00±61.27**	1.71±0.16**	5.54±0.57**
Evodiamine	Blank	6.04±0.54	466.02±72.08	1.00±0.09	15.00±0.46
	Control	6.67±1.32	614.08±52.56*	0.56±0.19**	14.77±1.15
	5μmol/L	7.07±0.38*	658.98±40.96**	0.50±0.16**	15.54±0.25
	6μmol/L	6.74±0.73	597.09±44.76*	0.57±0.17**	16.42±0.66*
	7μmol/L	6.22±1.15	612.86±52.77*	0.49±0.23**	16.42±0.87*

*P<0.05

**P<0.01 compared with the blank group. ^Δ^P<0.05, ^ΔΔ^P<0.01 compared with the control group, n = 4.

### The results of Western blot assay

**Fig 6:**
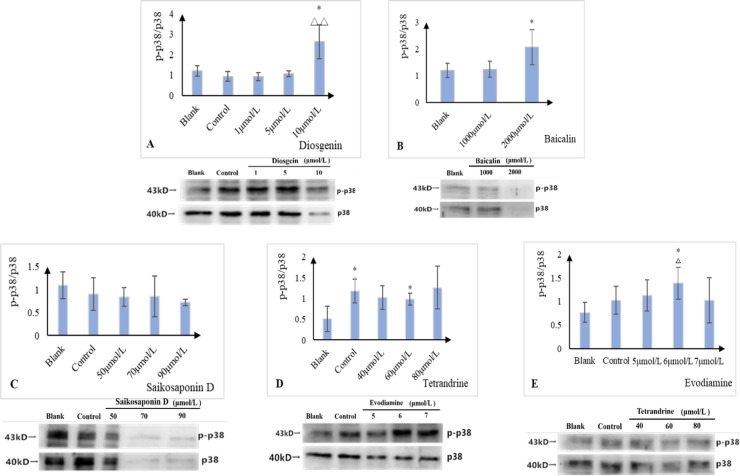
Effect of potential liver injury components on the expression of p38 protein.

According to the results of network pharmacology, we examined the expression levels of p38α. Antibodies was used as follows: p38α antibody (CST, 9218S), Phospho-p38 MAPK (Thr180/Tyr182) (D3F9) XP Rabbit mAb (CST, 4511S), IgG H&L (HRP) (Abcam, ab6721).As shown in Fig 6, the results of western blot indicated that the ratios of p-p38α/p38α expression were all increased in L-02 cells treated by 10μmol/L diosgein, 2000μmol/L baicalin, 60μmol/L terandrine and 6μmol/L evodiamine compared with the blank groups. While each dose in saikosaponin D group showed relatively weak signal intensities in the expression of p-p38α/p38α. The Specific specific composition information is was shown in S3 Table. Overall, the mechanism of liver injury influenced by diosgein, baicalin, terandrine and evodiamine in correlation with the western blot data was related to the increased expression of p38α. However, the mechanism of saikosaponin D causing liver injury still remained unclear.

**Fig 7:**
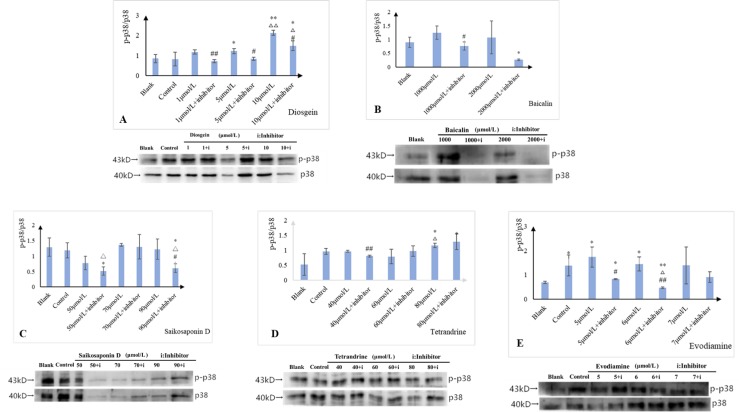
Effects of potential liver injury components on L-02 cells p38 protein expression activity with inhibitor.

To further and fully elucidating the mechanism of the liver injury caused by the representative compounds from urate-lowering Chinese herbs, we chose the p38α inhibitor, SB203580 to verify our findings. Seemingly, the ALT, AST, ALP, and LDH activities of each group decreased to varying degrees under the influence of inhibitor. The specific composition information was shown in S2 Table. Also, the result indicated that SB203580 could attenuate the component-induced cell damage of L-02 cells. Similarly, as shown in Fig 7, the ratios of p-p38α/p38α expression were all decreased in L-02 cells treated by the combination of components and SB203580 compared with the blank groups. Combined with changes in enzymatic indicators, it is suggested that the mechanism of the liver injury caused by the representative compounds from urate-lowering Chinese herbs may be implicated into the activation of p38α, by contrast, SB203580 may ameliorate L-02 cell injury induced by the representative components. The Specific specific composition information is was shown in S4 Table.

## Discussion

Current study, the approaches of the literature mining, network pharmacology, molecular docking, and cell experiment validation were undertaken to decipher the underlying drug-induced liver injury molecular mechanisms of urate-lowering Chinese herbs. Our study revealed that the representative compounds with liver injury from urate-lowering Chinese herbs included diosgenin, rutaecarpine, evodiamine, tetrandrine, saikosaponin D and baicalin, moreover, MAPK signaling pathway (p38) was the main action pathways closely related to liver injury for these compounds, and the mechanism of the liver injury induced by these compounds may be associated with promoting the expression of p38α.

Through the comprehensive searching of published experiment and literature data, we identified 171 Chinese herbs and TCM formulations that were beneficial for treating hyperuricemia, these Chinese herbs covered the categories of dampness-eliminating, blood-activating and stasis-removing, deficiency-supplementing and so on. Also, the nature of these herbs’ distribution was widespread, and they produced satisfactory therapeutic effects for different syndrome types of hyperuricemia clinically; however, there were ADR reports of liver injury for some urate-lowering Chinese herbs during long-term treatment of hyperuricemia. According to the pharmacological studies, as the a bisbenzylisoquinoline alkaloid isolated from the Chinese herb *Stephania tetrandra S*. *Moore*., trandrine-induced mitochondrial dysfunction and the sources of oxidative stress may contribute to the apoptosis of live cells, and the overexpression of CYP2E1 could enhance the tetrandrine-induced cytotoxicity [69–70]. In addition, the aqueous extract of *Evodiae fructus* could trigger the liver cell death signaling, and the hepatotoxicity mechanisms were related to the dehydrogenation of evodiamine and rutaecarpine that may cause toxicities through the formation of electrophilic intermediates via CYP3A4 inactivation [71–72]. To address the liver injury mechanisms of compounds from urate-lowering Chinese herbs, we collected and analyzed the research in the field of drug-induced liver injury, a total of 32 candidate compounds were obtained through. Several of the potential liver injury targets of active compounds from urate-lowering Chinese herbs were identified by means of network pharmacology, among them, the MAPK protein yielded the core target with the higher degree centrality in our research. Recent medical studies confirm that it plays the crucial roles in diverse developmental and physiological processes involving in the cell response to outside stimuli by activating and regulating the client protein, for example, it also has been proven that p38 is closely connected with the stress, physical and chemical reactions within the cells [73–75]. As the ubiquitously expressed prototype member of the MAPK family, the expression of p38α is abundant in most tissues in response to various extracellular stimuli in different organisms [76–77], p38α pathway is an essential protein of proliferation, differentiation, migration, survival, apoptosis, and autophagy of cells, the response to actin remodeling, angiogenesis and DNA damage has been highlighted over the last decade, hence, the p38α was selected as the protein receptor that might be associated with liver injury for molecular docking [78–79]. With regard to the normal liver cell, the p38 activation might connect with triptolide-induced hepatotoxicity in rats, and play an important role both in the hepatic expression of proinflammatory cytokines and development of inflammation-related liver damage [80–81]. In summary, the p38 protein possesses an interesting area of research in liver injury. Furthermore, according to the docking scores between 32 candidate compounds and p38, a total of 6 representative compounds (diosgenin, rutaecarpine, evodiamine, tetrandrine, saikosaponin D and baicalin) with a high affinity were selected to cell experiments validation subsequently.

We performed the cell experiment in L-02 cells to confirm and highlight the liver injury mechanism of 6 representative compounds, due to the L-02 cells are widely applied to investigate the underlying mechanism of hepatoprotective effects or cytotoxicity recently [82–83]. Remarkably, the experimental works and clinical reports have revealed that ALT, AST, LDH, ALP activity are increased in the condition of liver damage, among the liver injury markers, ALT and AST are probably the most commonly used in both clinical diagnosis and research involving liver damage for several decades [84–85]. After the investigation the activities of AST, ALT, LDH, and ALP under 6 representative compounds, the results revealed that these compounds could lead to a significant increase of AST, ALT, LDH, and ALP to cause L-02 cell damage. Besides, baicalin could inhibit the proliferation of L-02 cells instead of inducing the cell damage because it did not achieve the significant increase of AST, ALT, LDH and ALP the dose of 2000μmol/L. Furthermore, the results of western blot demonstrated that the mechanism of liver injury induced by diosgenin, evodiamine, tetrandrine, and baicalin was correlative with increase the expression of p-p38α/p38α. However, the expression of p-p38α/p38α under the varying dose of saikosaponin D was without significant change. It has been validated that Saikosaponin D may block PDGF-BB and TGF-β1-induced cell proliferation and migration, inhibit proliferation and activation of HSC-T6 via decreasing phosphorylation of p38 [86]. Taken together, Saikosaponin D mainly played an indirect role in the regulation of p38 expression through activating other pathways, this point reflects the complexity of pharmaceutical distribution and metabolize in vivo. Herein, it is hard to extract the protein in rutaecarpine group owing to the massive cell death caused by the cytotoxicity of rutaecarpine and solubilizers. Based on the experimental works and clinical reports, SB203580 was accepted as the pyridinyl imidazole inhibitor of p38 with its favorable selectivity, which controls the various inflammatory responses and cellular stresses [87–88]. Interestingly, according to modern pharmacological research, the local application of p38 agonist anisomycin increased new bone formation in distraction osteogenesis, whereas SB203580 has the promising activity to decrease it through inhibiting p38 [89]. And it is demonstrated that the novel mechanism of SB203580 is relative to activate directly AhR-induced Cyp1a1 gene expression in an AhR-dependent manner [90]. In this regard, the SB203580 at the dose of 20μmol/L was supplemented in cell culture to observe the levels of AST, ALT, LDH and ALP, and the expression of p-p38α/p38α; the relative results displayed that the liver injury mechanism of representative compounds with potential hepatotoxicity was closely related to p38 activation, SB203580 modulates the downstream signals to p38 and reduces drug-induced liver injury. Nevertheless, SB203580 could not reverse the process of cell damage induced by compounds with hepatotoxicity, it suggested that the cell damage was involved and cooperated with other pathways. Although the further animal studies in vivo and pharma-toxicology studies will be pivotal for supporting our findings, the results of present study provide an overview of the liver injury mechanism induced by Chinese herbs from the network perspective and bioinformatics approach, the research route, research method, and finding can be valuable and beneficial for the identification and development of compounds with hypotoxicity from Chinese herbs.

## Conclusions

Overall, based on the bioinformatics approaches to combine the network pharmacology, computer simulation and molecular biology experiments, our results demonstrated that the compounds with potential liver injury from urate-lowering Chinese herbs, diosgenin, rutaecarpine, evodiamine, tetrandrine, saikosaponin D and baicalin, could lead to the decline the survival rate of L-02 cell, increase the activities of AST, ALT, LDH and ALP cell-culture medium, enhance the expression of p-p38/p38, and the p38 inhibitor could achieve the trend of regulating and controlling liver injury. These research findings bring further support to the growing evidence that the mechanism of the liver injury induced by the compounds from urate-lowering Chinese herbs may be associated with the activation of p38α.

## Supporting information

S1 Table Effects of potential liver injury components on L-02 cells viability. *P<0.05, **P<0.01 compared with the control group, n = 6.
(PDF)

S2 Table ALT, AST, LDH, ALP enzyme activity of L-02 cells interfered with the potential liver injury components and inhibitor. *P<0.05, **P<0.01 compared with the blank group. ^Δ^P<0.05, ^ΔΔ^P<0.01 compared with the control group, n = 4.
(PDF)

S3 Table Expression of p-p38α/p38α in L-02 cells interfered with the potential liver injury components. *P<0.05, **P<0.01 compared with the blank group. Extract protein after 24 hours of incubation. Compared with the control group, ^Δ^P<0.05, ^ΔΔ^P<0.01, n = 4.
(PDF)

S4 Table Expression of p-p38α/p38α in L-02 cells interfered with the potential liver injury components and inhibitor. *P<0.05, **P<0.01 compared with the blank group. Extract protein after 24 hours of incubation. Compared with the control group, ^Δ^P<0.05, ^ΔΔ^P<0.01, n = 4.
(PDF)
