# Correction: Molecular mechanisms involved in drug-induced liver injury caused by urate-lowering Chinese herbs: A network pharmacology study and biology experiments

**DOI:** 10.1371/journal.pone.0229032

**Published:** 2020-03-13

**Authors:** Fan Li, Yi-Zhu Dong, Dan Zhang, Xiao-Meng Zhang, Zhi-Jian Lin, Bing Zhang

The Funding statement is incorrect. The correct Funding statement is as follows: This research is supported by the National Natural Science Fund of China (No.81874349).
