# Supplementary material for: Molecular mechanisms involved in drug-induced liver injury caused by urate-lowering Chinese herbs: A network pharmacology study and biology experiments
Source: PLoS One. 2019 May 29;14(5):e0216948. doi: 10.1371/journal.pone.0216948 (PMC6541264; doi:10.1371/journal.pone.0216948)
Supplement: S1 Table — *P<0.05, **P<0.01 compared with the control group, n = 6. (PDF) [file pone.0216948.s001.pdf]

Supplementary Table 1 Effects of potential liver injury components on L-02 cells viability

**Group**	**Concentration (μmol/L)**	**Absorbance (A)**	cells viabilityrate (%)
Diosgenin	0	0.34±0.04	100.00±0.00
	1	0.34±0.03	101.29±15.44
	5	0.08±0.02**	24.11±4.32**
	10	0.03±0.01**	7.75±2.50**
Baicalin	0	0.38±0.04	100.00±0.00
	1000	0.59±0.04	153.52±21.12
	2000	0.38±0.06	98.58±16.48
Saikosaponin D	0	0.66±0.21	100.00±0.00
	50	0.74±0.19	115.78±18.15*
	70	0.43±0.12*	59.51±31.32**
	90	0.40±0.13**	62.67±10.09**
Tetrandrine	0	0.30±0.01	100.00±0.00
	40	0.31±0.02	101.33±9.72
	60	0.28±0.01**	91.79±4.63**
	80	0.25±0.03**	83.34±5.22**
Rutaecarpine	0	0.36±0.05	100.00±0.00
	5	0.33±0.05	92.66±3.74**
	10	0.29±0.05*	81.32±6.29**
	15	0.29±0.05*	80.48±3.90**
Evoidiamine	0	0.31±0.05	100.00±0.00
	5	0.27±0.03	85.02±15.70*
	6	0.23±0.01*	50.70±37.42**
	7	0.22±0.06*	66.87±11.58**

$^{*}P<0.05$, $^{**}P<0.01$ compared with the control group , n=6.
