# Supplementary material for: Molecular mechanisms involved in drug-induced liver injury caused by urate-lowering Chinese herbs: A network pharmacology study and biology experiments
Source: PLoS One. 2019 May 29;14(5):e0216948. doi: 10.1371/journal.pone.0216948 (PMC6541264; doi:10.1371/journal.pone.0216948)
Supplement: S2 Table — *P<0.05, **P<0.01 compared with the blank group. ΔP<0.05, ΔΔP<0.01 compared with the control group, n = 4. (PDF) [file pone.0216948.s002.pdf]

Supplementary Table 2 ALT, AST, LDH, ALP enzyme activity of L-02 cells interfered with the potential liver injury components and inhibitor

	**Group**	ALT（U/L）	AST（U/L）	LDH（U/L）	ALP (King's unit /100ml)
Diosgenin	Blank	16.25±0.43	5.81±0.23	351.24±2.41	1.70±0.23
	Control	16.85±0.75	7.28±1.21*	533.67±35.52**	1.79±0.04
	1μmol/L	16.93±0.44	7.53±0.61*	537.08±21.70**	1.47±0.40
	1μmol/L+inhibitor	15.32±0.82#	6.71±0.92	414.32±18.20**△△##	1.46±0.08**△△
	5μmol/L	16.16±0.67	6.88±0.50*	530.26±19.29**	2.63±0.21**△△
	5μmol/L+inhibitor	17.04±2.11	7.03±1.41	409.21±29.24*△△##	3.84±0.07**△△##
	10μmol/L	15.91±0.48	7.53±0.11**	603.58±19.14**△	4.88±0.44**△△
	10μmol/L+inhibitor	17.63±1.61	7.53±0.50**	509.80±9.65**##	4.31±0.15**△△
Baicalin	Blank	1.55±0.19	1.52±0.80	473.93±13.40	0.90±0.09
	1000μmol/L	2.24±0.30*	4.45±1.09*	699.84±33.81**	0.86±0.06**
	1000μmol/L+inhibitor	1.88±0.20*	4.12±0.26**	778.83±14.65**##	0.70±0.05**#
	2000μmol/L	2.01±0.20*	4.48±0.13**	840.44±32.45**	0.92±0.13**
	2000μmol/L+inhibitor	2.27±0.32*	4.38±0.84**	981.04±3.87**##	0.85±0.06**
Saikosaponin D	Blank	14.99±1.13	6.34±0.68	365.20±32.23	1.30±0.03
	Control	15.40±0.43	6.85±0.67	578.81±27.67**	1.16±0.11
	50μmol/L	15.91±1.04	8.27±0.99*	580.53±15.98**	1.22±0.23
	50μmol/L+inhibitor	20.99±0.56**△△##	14.88±1.00**△△##	1245.48±30.43**△△##	1.08±0.07**
	70μmol/L	24.16±1.23**△△	14.64±0.51**△△##	1493.54±44.05**△△	1.18±0.10
	70μmol/L+inhibitor	21.35±0.78**△△##	13.31±2.12**△△	1245.48±91.38**△△#	0.89±0.17*
	90μmol/L	22.65±1.08**△△	16.37±0.40**△△	1529.72±25.67**△△	1.03±0.08**
	90μmol/L+inhibitor	19.03±1.58**△△##	8.75±1.65**△△##	1052.54±49.99**△△##	1.59±0.10**△△##
Tetrandrine	Blank	14.36±0.82	3.37±0.21	553.67±35.66	0.88±0.08
	Control	15.00±0.60	4.52±0.80	547.22±24.69	1.08±0.10*
	40μmol/L	16.55±0.71*△	4.36±0.35*	713.48±49.44*△△	0.82±0.06△
	40μmol/L+inhibitor	15.73±0.00*	5.01±0.41**	694.11±30.20**△△	0.82±0.06△
	60μmol/L	16.39±0.81*	5.64±0.56**	760.29±34.47**△△	0.82±0.06△
	60μmol/L+inhibitor	15.49±1.00	4.92±0.75*	721.55±32.36**△△	0.75±0.10△
	80μmol/L	17.45±0.35**△△	5.16±0.75*	769.98±58.25**△△	0.97±0.03
	80μmol/L+inhibitor	16.88±0.12**△△##	3.63±2.20	745.76±31.63**△△	0.92±0.06
Rutaecarpine	Blank	1.99±0.18	2.66±0.99	533.02±13.69	1.02±0.10
	Control	5.86±0.09**	9.42±1.62**	1040.57±20.62	1.90±0.50*
	5μmol/L	6.00±0.43**	10.33±0.74**	1105.81±85.95**	1.63±0.03**
	5μmol/L+inhibitor	5.01±0.13**△△#	9.25±1.09**	1193.32±3.90**	1.13±0.12△#
	10μmol/L	5.67±0.12**	9.81±0.74**	1155.13±35.72**△△	1.07±0.14△
	10μmol/L+inhibitor	5.63±0.09**△	10.52±0.97**	1217.18±41.25**△△	1.39±0.28**#
	15μmol/L	5.54±0.57**	10.02±1.63**	1031.03±33.98**	1.77±0.13**
	15μmol/L+inhibitor	5.29±0.38**	8.96±1.37**	1061.26±48.73**	1.10±0.20#
Evoidiamine	Blank	14.76±0.24	5.82±0.44	425.57±19.55	0.96±0.07
	Control	14.77±1.15	6.67±1.32	585.76±21.83**	0.65±0.12*
	5μmol/L	15.54±0.25*	7.28±0.12**	681.23±16.02**△△	0.56±0.14*

5μmol/L+inhibitor	15.81±0.86	7.11±0.67*	689.32±39.64$^{**\triangle}$	0.44±0.14**
6μmol/L	16.42±0.66*	7.02±0.64*	614.89±37.46$^{**\triangle\triangle}$	0.62±0.19*
6μmol/L+inhibitor	16.42±0.45	6.51±0.12*	700.65±16.02$^{**\triangle\triangle\#\#}$	0.51±0.17*
7μmol/L	16.86±0.25$^{**\triangle}$	6.50±1.20	629.45±51.12**	0.60±0.17*
7μmol/L+inhibitor	15.63±0.99	6.76±0.94	726.54±47.95$^{**\triangle\triangle}$	0.70±0.10*

*P<0.05, **P<0.01 compared with the blank group. $^{\Delta}$P<0.05, $^{\Delta\Delta}$P<0.01 compared with the control group, n=4.
