# Supplementary material for: Molecular mechanisms involved in drug-induced liver injury caused by urate-lowering Chinese herbs: A network pharmacology study and biology experiments
Source: PLoS One. 2019 May 29;14(5):e0216948. doi: 10.1371/journal.pone.0216948 (PMC6541264; doi:10.1371/journal.pone.0216948)
Supplement: S3 Table — *P<0.05, **P<0.01 compared with the blank group. Extract protein after 24 hours of incubation. Compared with the control group, ΔP<0.05, ΔΔP<0.01, n = 4. (PDF) [file pone.0216948.s003.pdf]

Supplementary Table 3 Expression of p-p38α/p38α in L-02 cells interfered with the potential liver injury components

Group		p-p38α/p38α
Diosgenin	Blank	1.20±0.25
	Control	0.92±0.24
	1μmol/L	0.93±0.20
	5μmol/L	1.06±0.14
	10μmol/L	2.63±0.83**△△
Baicalin	Blank	1.20±0.27
	1000μmol/L	1.24±0.29
	2000μmol/L	2.06±0.66*
Saikosaponin D	Blank	0.10±0.29
	Control	0.91±0.35
	50μmol/L	0.84±0.20
	70μmol/L	0.86±0.44
	90μmol/L	0.72±0.07
Tetrandrine	Blank	0.51±0.31
	Control	1.18±0.29*
	40μmol/L	1.03±0.29
	60μmol/L	0.98±0.14*
	80μmol/L	1.26±0.52
Evodiamine	Blank	0.77±0.21
	Control	1.03±0.30
	5μmol/L	1.13±0.33
	6μmol/L	1.39±0.34*
	7μmol/L	1.03±0.48

*P<0.05, **P<0.01 compared with the blank group. Extract protein after 24 hours of incubation. Compared with the control group, △P<0.05, △△P<0.01, n=4.
