# Supplementary material for: Molecular mechanisms involved in drug-induced liver injury caused by urate-lowering Chinese herbs: A network pharmacology study and biology experiments
Source: PLoS One. 2019 May 29;14(5):e0216948. doi: 10.1371/journal.pone.0216948 (PMC6541264; doi:10.1371/journal.pone.0216948)
Supplement: S4 Table — *P<0.05, **P<0.01 compared with the blank group. Extract protein after 24 hours of incubation. Compared with the control group, ΔP<0.05, ΔΔP<0.01, n = 4. (PDF) [file pone.0216948.s004.pdf]

Supplementary Table 4 Expression of p-p38α/p38α in L-02 cells interfered with the potential liver injury components and inhibitor

Group		p-p38α/p38α
Diosgenin	Blank	0.84±0.21
	Control	0.82±0.33
	1μmol/L	1.17±0.1
	1μmol/L+inhibitor	0.72±0.07##
	5μmol/L	1.22±0.12*
	5μmol/L+inhibitor	0.83±0.08#
	10μmol/L	2.13±0.13**△△
	10μmol/L+inhibitor	1.49±0.24*△#
Baicalin	Blank	0.9±0.18
	1000μmol/L	1.25±0.24
	1000μmol/L+inhibitor	0.76±0.15#
	2000μmol/L	1.08±0.6
	2000μmol/L+inhibitor	0.26±0.03*
Saikosaponin D	Blank	1.29±0.3
	Control	1.19±0.24
	50μmol/L	0.78±0.21
	50μmol/L+inhibitor	0.52±0.13*△
	70μmol/L	1.37±0.05
	70μmol/L+inhibitor	1.3±0.39
	90μmol/L	1.22±0.33
	90μmol/L+inhibitor	0.61±0.14*△#
Tetrandrine	Blank	0.53±0.36
	Control	0.96±0.1
	40μmol/L	0.97±0.03
	40μmol/L+inhibitor	0.81±0.03##
	60μmol/L	0.79±0.24
	60μmol/L+inhibitor	0.98±0.17
	80μmol/L	1.17±0.07*△
	80μmol/L+inhibitor	1.28±0.26*
Evodiamine	Blank	0.69±0.05
	Control	1.39±0.43*
	5μmol/L	1.74±0.42*
	5μmol/L+inhibitor	0.82±0.02*#
	10μmol/L	1.45±0.29*
	10μmol/L+inhibitor	0.47±0.03**△##
	15μmol/L	1.39±0.77
	15μmol/L+inhibitor	0.91±0.22

*P<0.05, **P<0.01 compared with the blank group. Extract protein after 24 hours of incubation. Compared with the control group, $^{\Delta}$P<0.05, $^{\Delta\Delta}$P<0.01, n=4.
